# Yeast identification, antifungal susceptibility testing, and *Candida auris* screening practices at acute care hospitals and long-term acute care hospitals, United States

**DOI:** 10.1128/jcm.00091-26

**Published:** 2026-04-20

**Authors:** Jeremy A. W. Gold, Kaitlin Benedict, Kathryn A. Haass, Hemjot Kaur, Pranjal Muthe, Beth A. Bouwkamp, Meghan Lyman, Mitsuru Toda, Tiffany Rivers, Shawn R. Lockhart

**Affiliations:** 1 Mycotic Diseases Branch, Centers for Disease Control and Prevention 1242, Atlanta, Georgia, USA; 2 Division of Healthcare Quality Promotion, Centers for Disease Control and Prevention 549360 , Atlanta, Georgia, USA; 3 Leidos Inc 399170, Atlanta, Georgia, USA; 4 CACI International Inc 118774 , Atlanta, Georgia, USA; University of Calgary, Calgary, Alberta, Canada

**Keywords:** antifungal susceptibility testing, *Candida auris*, healthcare-associated infections, long-term acute care hospitals, MALDI-TOF MS, National Healthcare Safety Network, species identification, yeast identification

## Abstract

Accurate species identification and antifungal susceptibility testing (AFST) are critical for detection and clinical management of emerging antifungal-resistant yeasts such as *Candida auris. C. auris* colonization screening is essential for preventing its spread. However, nationally representative data on these practices are limited. We analyzed data from 5,333 acute care hospitals (ACHs) and 359 long-term acute care hospitals (LTACHs) enrolled in Centers for Disease Control and Prevention (CDC)’s National Healthcare Safety Network (NHSN), Patient Safety Component (PSC), specifically facility responses to the 2024 NHSN survey. Nearly all hospitals (≥99%) reported having yeast identification performed, most frequently at on-site laboratories (ACH: 41%, LTACH: 32%) or affiliated medical center laboratories (ACH: 28%, LTACH: 30%). Matrix-assisted laser desorption/ionization time-of-flight mass spectrometry (MALDI-TOF MS) was the predominant method used (ACH: 71%, LTACH: 63%); use of chromogenic agar to differentiate *Candida* isolates was less common (ACH: 27%, LTACH: 32%). Most hospitals (≥96% of ACHs and LTACHs) reported having AFST performed, primarily at a commercial laboratory (ACH: 55%, LTACH: 49%) rather than on-site or at an affiliated facility. Routine *C. auris* screening was less common (ACHs: 21%, LTACHs: 33%). Among those, 86% of LTACHs and 3% of ACHs reported universal admission screening. These data provide updated benchmarks of yeast identification, AFST, and *C. auris* screening in U.S. hospitals. Reliance on off-site AFST might delay results, potentially impairing timely treatment decisions and step-down therapy. National benchmark data on *C. auris* screening could help guide efforts to improve containment strategies.

**IMPORTANCE:**

Accurate yeast identification and antifungal susceptibility testing (AFST) are important for detecting and managing drug-resistant fungi, particularly *Candida auris*. Screening patients for *C. auris* also helps prevent spread, but national data on these practices are limited. We analyzed data from 5,333 acute care hospitals (ACHs) and 359 long-term acute care hospitals (LTACHs) reporting to Centers for Disease Control and Prevention (CDC)’s National Healthcare Safety Network, Patient Safety Component (PSC). Nearly all hospitals reported having yeast identification and AFST available, although these services were often performed at off-site or commercial laboratories, which may delay results and affect timely treatment decisions. Most hospitals used matrix-assisted laser desorption/ionization time-of-flight mass spectrometry (MALDI-TOF MS) for yeast identification, while fewer routinely used chromogenic agar to differentiate *Candida* isolates, representing a potential opportunity to strengthen laboratory practices. Routine *C. auris* screening was reported by 21% of ACHs and 33% of LTACHs. These findings provide national benchmarks that may help guide efforts to improve detection and containment of *C. auris*.

## INTRODUCTION

In the United States, *Candida* species are the most frequent cause of fungal infections, and rising antifungal resistance poses clinical and public health concerns (1–3). Candidemia is the second-most common healthcare-associated bloodstream infection, associated with a ~33% in-hospital mortality rate (3, 4). Infectious Diseases Society of America (IDSA) guidelines recommend identifying all *Candida* bloodstream isolates to the species level, performing azole susceptibility testing for all bloodstream and other clinically relevant *Candida* isolates, and considering echinocandin susceptibility testing for patients with prior echinocandin receipt or infections with *C. glabrata* or *C. parapsilosis* (5). Accurate and timely species identification and antifungal susceptibility testing (AFST) are critical to guide clinical management and detect emerging resistant pathogens (6, 7).

However, national data on yeast identification practices in U.S. hospitals are lacking, and national AFST practices were characterized among acute care hospitals (ACHs) nearly a decade ago (8) before the global emergence of *Candida auris* and the rising predominance of non-*albicans Candida* species (2, 3). Data are particularly lacking from long-term acute care hospitals (LTACHs), which have been disproportionately affected by *C. auris* outbreaks (2).

*C. auris* poses an urgent public health threat because of its frequent multidrug resistance, ability to cause life-threatening infections, potential for asymptomatic colonization of patients, and persistence in healthcare environments, which facilitates rapid spread in these settings (2, 9, 10). Colonization screening is a key component of preventing spread as early detection can prompt rapid implementation of infection prevention and control measures (2). Centers for Disease Control and Prevention (CDC) guidelines recommend conducting *C. auris* colonization screenings based on local epidemiology, patient characteristics, and facility-specific risk factors (https://www.cdc.gov/hai/mdro-guides/prevention-strategy.html). However, nationally representative data on hospital screening practices for *C. auris* are limited (10).

To address these gaps, we described yeast identification, AFST practices, and *C. auris* screening practices among ACHs and LTACHs enrolled in the CDC’s National Healthcare Safety Network (NHSN), Patient Safety Component (PSC). NHSN is the most widely used healthcare-associated infection tracking system in the United States (https://www.cdc.gov/nhsn/about-nhsn/index.html), and the Patient Safety Component (PSC) monitors process measures and events related to medical devices, surgical procedures, antimicrobial use, and multidrug-resistant organisms (https://www.cdc.gov/nhsn/psc/index.html).

## MATERIALS AND METHODS

Facilities enrolled in the NHSN PSC are required to submit a PSC Annual Survey for their facility type (i.e., hospital, LTACH, or Inpatient Rehabilitation Facility [IRF]) at the beginning of each year, reporting data from the previous year, hereafter referred to as the survey year. The PSC Annual Hospital, LTACH, and IRF Surveys collect facility-level data about facility characteristics, standard operations, and infection prevention practices, including yeast identification, AFST practices, and *C. auris* screening practices (https://www.cdc.gov/nhsn/forms/57.103_pshospsurv_blank.pdf, https://www.cdc.gov/nhsn/forms/57.150_LTACFacSurv_BLANK.pdf). The NHSN application incorporates standardized business rules and automated data checks for PSC Annual Surveys to ensure completeness, consistency, and data quality.

We analyzed NHSN PSC Annual Survey data for survey year 2024 for both ACH and LTACH. IRF surveys were excluded from the analysis. Facilities were considered active if they submitted a PSC Annual Facility Survey in 2024 or 2023. The number of enrolled facilities is higher and includes facilities that have not reported a survey in recent years. We examined responses regarding yeast identification practices (matrix-assisted laser desorption/ionization time-of-flight mass spectrometry [MALDI-TOF MS], chromogenic agar, molecular methods, and referral testing), AFST practices (methods used, antifungals tested, and triggers for testing), and *C. auris* screening (whether routine screening is performed, screening triggers, and testing methods used) (https://www.cdc.gov/nhsn/psc/locations.html). We performed descriptive analyses, stratifying by ACH and LTACH. Responses were excluded from the denominator for analyses specific to a given survey item if hospitals indicated they did not have yeast identification or AFST performed, marked the item as “not applicable,” or had missing data for that item. These exclusions were applied at the item level; facilities were included in other analyses for which they provided applicable data. Data were frozen on 16 May 2025 and analyzed using SAS version 9.4 software (SAS Institute, Cary, North Carolina).

## RESULTS

### NHSN Annual Survey

For the 2024 survey year, 5,333 ACHs and 359 LTACHs submitted PSC Annual Surveys and comprised the analytic sample. This represents greater than 95% of active facilities in each setting.

### Yeast identification practices

Overall, 5,267 (99%) ACHs and 358 (>99%) LTACHs reported having yeast identification performed. Of those, yeast identification was performed most frequently at on-site laboratories (ACH: 41%, LTACH: 32%), followed by affiliated medical center laboratories (ACH: 28%, LTACH: 30%) and commercial referral laboratories (ACH: 25%, LTACH: 26%) (Table 1). The predominant yeast identification method was MALDI-TOF MS (ACH: 71%, LTACH: 63%), with approximately one-third using it exclusively (ACH: 36%, LTACH: 29%). The next most common method was VITEK 2 (ACH: 37%, LTACH: 47%), with fewer facilities using it exclusively (ACH: 10%, LTACH: 14%). At fewer than one-third of facilities (ACH: 27%, LTACH: 32%), testing routinely involved using chromogenic agar to identify or differentiate *Candida* isolates.

**TABLE 1:** Yeast identification at facilities participating in the NHSN Annual Facility Survey, 2024 Survey Year^*a*^

Characteristic	Total		ACH		LTACH	
	*n* = 5,625	%	*n* = 5,267	%	*n* = 358	%
Where is yeast identification performed for specimens collected at your facility?^*b*^						
Affiliated medical center	1,556	28%	1,449	28%	107	30%
Commercial referral laboratory	1,387	25%	1,293	25%	94	26%
On-site laboratory	2,270	40%	2,157	41%	113	32%
Other^*c*^	412	7%	368	7%	44	12%
Which of the following methods are used for yeast identification?^*d*^						
MALDI-TOF MS System	3,990	71%	3,765	71%	225	63%
MALDI-TOF MS System (Vitek MS)	2,165	38%	2,027	38%	138	39%
MALDI-TOF MS System (Bruker Biotyper)	1,893	34%	1,804	34%	89	25%
Vitek 2	2,132	38%	1,963	37%	169	47%
BD Phoenix	166	3%	152	3%	14	4%
MicroScan	241	4%	215	4%	26	7%
Non-automated Manual Kit (for example, API 20C, RapID, Germ Tube, and PNA-FISH)	683	12%	642	12%	41	11%
DNA sequencing	191	3%	171	3%	20	6%
Other	660	12%	633	12%	27	8%
Does the laboratory routinely use chromogenic agar for the identification or differentiation of *Candida* isolates?						
Yes	1,527	27%	1,414	27%	113	32%
No	3,740	66%	3,528	67%	212	59%
Unknown	358	6%	325	6%	33	9%
*Candida* isolated from which of the following body sites are usually fully identified to the species level? (select all that apply)						
Blood	5,257	93%	4,912	93%	345	96%
Other normally sterile body sites (for example, CSF)	5,021	89%	4,720	90%	301	84%
Urine	3,039	54%	2,823	54%	216	60%
Respiratory	2,289	41%	2,121	40%	168	47%
Other	1,282	23%	1,195	23%	87	24%
None	174	3%	170	3%	4	1%
Does the laboratory employ any polymerase chain reaction (PCR) molecular tests to identify *Candida* from blood specimens?						
Yes	2,648	47%	2,494	47%	154	43%
No	2,699	48%	2,526	48%	173	48%
Unknown	278	5%	247	5%	31	9%
If yes, which PCR molecular tests are used to identify *Candida* from blood specimens? (select all that apply)						
T2Candida Panel	85	3%	73	3%	12	8%
BioFire BCID	1,886	71%	1,794	72%	92	60%
GenMark ePlex BCID	286	11%	274	11%	12	8%
Other	421	16%	392	16%	29	19%
Unknown	56	2%	43	2%	13	8%
If yes and you get a positive result, does this lab culture the blood to obtain an isolate?						
Yes, always	2,387	90%	2,266	91%	121	79%
Yes, with clinical order	102	4%	88	4%	14	9%
No	53	2%	48	2%	5	3%
Unknown	106	4%	92	4%	14	9%

^*a*^ ACH = acute care hospital, LTACH = long-term acute care hospital, MALDI-TOF MS = matrix-assisted laser desorption/ionization time-of-flight mass spectrometry, PCR = polymerase chain reaction, PNA-FISH = peptide nucleic acid fluorescent *in situ* hybridization, and CSF = cerebrospinal fluid.

^*b*^ Facilities that reported not having yeast identification performed (ACH: n = 10, LTACH: n = 0) or indicated that it was “not applicable” (ACH: n = 56, LTACH: n = 1) were excluded. Among the 5,267 included ACHs, the most common types were general hospital (64%), critical access hospital (24%), psychiatric hospital (3%), veterans affairs hospital (2%), and children's hospital (2%). Among the 66 excluded ACHs, the most common types were critical access hospital (42%), general hospital (35%), and psychiatric hospital (17%).

^*c*^ Other local/regional, non-affiliated reference laboratory.

^*d*^ A total of 1,915 (36%) ACH and 104 (29%) LTACH had MALDI-TOF MS only (no other method). A total of 551 (10%) ACHs and 51 (14%) LTACH had Vitek 2 only.

For most facilities, *Candida* was usually identified to the species level for specimens from blood (ACH: 93%, LTACH: 96%) and other normally sterile body sites (ACH: 90%, LTACH: 84%); this percentage was lower for urine (ACH: 54%, LTACH: 60%) and respiratory (ACH: 40%, LTACH: 47%) specimens. PCR molecular tests to identify *Candida* from blood specimens were used for less than half (ACH: 47%, LTACH: 43%); of those, BioFire Blood Culture Identification Panel (BCID) (bioMérieux, Marcy-l'Étoile, France) was most frequently used (ACH: 72%, LTACH: 60%). Among facilities using PCR, most reported that blood was always cultured following a positive PCR result to obtain an isolate (ACH: 91%, LTACH: 79%), consistent with continued use of culture-based identification.

### AFST practices

Overall, 5,142 (96%) ACHs and 348 (97%) LTACHs reported having AFST performed. Of those, AFST was most often performed at a commercial laboratory (ACH: 55%, LTACH: 49%) (Table 2). Fewer reported performing AFST at an affiliated medical center (ACH: 17%, LTACH: 21%) or an on-site laboratory (ACH: 15%, LTACH: 13%). The most frequently used AFST method (excluding for amphotericin B) was YeastOne (Trek Diagnostic Systems, Cleveland, United States) (ACH: 33%, LTACH: 31%), followed by broth microdilution with laboratory-developed plates (ACH: 19%, LTACH: 18%) or Vitek 2 (bioMérieux, Marcy-l'Étoile, France) (ACH: 17%, LTACH 21%); approximately one-quarter of facilities answered that the AFST method used was unknown (ACH: 28%, LTACH 25%). Most facilities performed AFST for fluconazole (ACH: 75%, LTACH: 75%), voriconazole (ACH: 69%, LTACH: 64%), micafungin (ACH: 68%, LTACH: 66%), and caspofungin (ACH: 63%, LTACH: 59%). At most facilities, AFST was performed automatically (without a clinician’s order) on blood isolates (ACH: 59%, LTACH: 55%) and isolates from other normally sterile (ACH: 52%, LTACH: 47%) body sites; <10% automatically performed AFST on urine, respiratory, or other specimen types.

**TABLE 2:** AFST at facilities participating in the NHSN Annual Facility Survey, 2024 Survey Year^*a*^

Characteristic	Total		ACH		LTACH	
	*n* = 5,490	%	*n* = 5,142	%	*n* = 348	%
Where is AFST identification performed for specimens collected at your facility?^*b*^						
Affiliated medical center	943	17%	870	17%	73	21%
Commercial referral laboratory	3,011	55%	2,839	55%	172	49%
On-site laboratory	840	15%	794	15%	46	13%
Other^*c*^	696	13%	639	12%	57	16%
What methods are used for AFST, excluding amphotericin B?^*d*^						
Broth microdilution with laboratory-developed plates	1,056	19%	993	19%	63	18%
YeastOne (Thermo Scientific SensiTitre)	1,805	33%	1,697	33%	108	31%
Gradient diffusion (E test)	267	5%	250	5%	17	5%
Vitek (bioMérieux)	969	18%	897	17%	72	21%
Other	417	8%	397	8%	20	6%
Unknown	1,503	27%	1,417	28%	86	25%
What methods are used for AFST of amphotericin B?^*d*^						
Broth microdilution with laboratory-developed plates	1,026	19%	962	19%	64	18%
YeastOne (Thermo Scientific SensiTitre)	1,229	22%	1,147	22%	82	24%
Gradient diffusion (E test)	144	3%	133	3%	11	3%
Vitek (bioMerieux)	268	5%	246	5%	22	6%
Other	1,185	22%	1,128	22%	57	16%
Unknown	1,854	34%	1,732	34%	122	35%
AFST is performed for which of the following antifungal drugs?^*d*^						
Fluconazole	4,101	75%	3,841	75%	260	75%
Voriconazole	3,785	69%	3,564	69%	221	64%
Itraconazole	2,831	52%	2,681	52%	150	43%
Posaconazole	2,354	43%	2,226	43%	128	37%
Micafungin	3,728	68%	3,499	68%	229	66%
Anidulafungin	2,276	41%	2,153	42%	123	35%
Caspofungin	3,440	63%	3,234	63%	206	59%
Amphotericin B	2,660	48%	2,500	49%	160	46%
Flucytosine	2,157	39%	2,019	39%	138	40%
Other	1,015	18%	959	19%	56	16%
Unknown	1,105	20%	1,035	20%	70	20%
AFST is performed automatically on which fungal isolates?^*d*^						
Blood	3,213	59%	3,023	59%	190	55%
Other normally sterile body sites (for example, CSF)	2,843	52%	2,680	52%	163	47%
Urine	286	5%	253	5%	33	9%
Respiratory	224	4%	195	4%	29	8%
Other	168	3%	159	3%	9	3%
AFST is performed with a clinician’s order on which fungal isolates?^*d*^						
Blood	1,618	29%	1,497	29%	121	35%
Other normally sterile body sites (for example, CSF)	1,960	36%	1,818	35%	142	41%
Urine	4,146	76%	3,902	76%	244	70%
Respiratory	3,913	71%	3,678	72%	235	68%
Other	747	14%	682	13%	65	19%
Is this laboratory developing antibiograms or other reports to track susceptibility trends for *Candida* spp. isolates tested in this laboratory?						
Yes	5,161	94%	4,842	94%	319	92%
No	329	6%	300	6%	29	8%

^*a*^ AFST = antifungal susceptibility testing, ACH = acute care hospital, LTACH = long-term acute care hospital, and CSF = cerebrospinal fluid.

^*b*^ Facilities that reported not having AFST performed (ACH: n = 66, LTACH: n = 1) or stated that it was “not applicable” (ACH: n = 125, LTACH: n = 10) were excluded. Among the 5,142 included ACHs, the most common types were general hospital (65%), critical access hospital (24%), psychiatric hospital (3%), veterans affairs hospital (2%), and children's hospital (2%). Among the 191 excluded ACHs, the most common types were general hospital (44%), critical access hospital (39%), and psychiatric hospital (10%).

^*c*^ Other local/regional, non-affiliated reference laboratory.

^*d*^ Select all that apply.

### *C. auris* screening

Data on whether routine screening testing for *C. auris* was performed were available for 5,323 (>99%) ACHs and 359 (100%) LTACHs. Of those, 21% (1,096/5,323) of ACHs and 33% (118/359) of LTACHs reported such testing (Table 3). For both hospital types, PCR was the predominant method (ACH: 57%, LTACH: 73%), followed by culture-based approaches (ACH: 47%, LTACH: 32%). ACHs most often routinely screened patients at admission if they were “high risk” (76%), epidemiologically linked patients (59%), or admitted from LTACHs or long-term care facilities (54%) but infrequently reported screening of all patients on admission (3%). LTACHs frequently reported screening for all patients upon admission (86%).

**TABLE 3:** *C. auris* screening at facilities participating in the NHSN Annual Facility Survey, 2024 Survey Year^*a*^

Characteristic	Total^*b*^		ACH		LTACH	
	*n* = 5,682	%	*n* = 5,323	%	*n* = 359	%
Does the facility routinely perform screening testing (culture or non-culture) for *C. auris*? This includes screening for patients at your facility performed by public health laboratories and commercial laboratories.^*c*^						
Yes	1,214	21%	1,096	21%	118	33%
No	4,468	79%	4,227	79%	241	67%
If yes, in which situations does the facility routinely perform screening testing for *C. auris*?^*d*^						
Surveillance testing at admission for all patients	135	11%	33	3%	102	86%
Surveillance testing of epidemiologically linked patients of newly identified *C. auris* patients (for example, point prevalence surveys in response to a case and patients in the same room or unit as a case)	682	56%	642	59%	40	34%
Surveillance testing at admission of high-risk patients	838	69%	831	76%	7	6%
Patients admitted from long-term acute care or long-term care facilities	599	49%	595	54%	4	3%
Patients with recent (for example, within 6 months) overnight hospital stay outside the United States	492	41%	490	45%	2	2%
Patients admitted to high-risk settings (for example, ICU)	152	13%	148	14%	4	3%
Other high-risk patients	397	33%	394	36%	3	3%
Surveillance testing of all patients in the facility or in a specific high-risk setting (for example, ICU) at prespecified intervals (for example, weekly point prevalence survey)	51	4%	35	3%	16	14%
Other	98	8%	88	8%	10	8%
If yes, what method is routinely used by the lab conducting *C. auris* testing of screening swabs from your facility?						
Culture-based methods	555	46%	517	47%	38	32%
PCR	711	59%	625	57%	86	73%
Other	71	6%	66	6%	5	4%

^*a*^ ACH = acute care hospital, LTACH = long-term acute care hospital, PCR = polymerase chain reaction, and ICU = intensive care unit.

^*b*^ Overall, 10 ACHs and 0 LTACHs were missing data on whether the facility routinely performed screening testing for *C. auris*; these responses were excluded.

^*c*^ Among the 1,096 ACHs that perform* C. auris* screening, the most common types were general hospital (81%), critical access hospital (10%), children's hospital (3%), veterans affairs hospital (2%), and psychiatric hospital (2%).

^*d*^ Select all that apply.

## DISCUSSION

Our analysis provides the first national description of yeast identification practices in U.S. ACHs and LTACHs. Encouragingly, the vast majority of facilities reported having *Candida* identified to the species level for bloodstream and other normally sterile site isolates, which is consistent with IDSA guidelines (5). Species-level identification was substantially less common for urine and respiratory specimens compared with bloodstream and other sterile site isolates. Although yeast recovered from nonsterile sites often represents colonization, species-level identification in selected clinical contexts might help monitor emerging resistance patterns. Additional studies could improve understanding of the clinical impact of species-level identification for non-sterile site isolates. MALDI-TOF MS was the predominant identification method (ACH: 71%, LTACH: 63%), reflecting substantial progress toward faster, more accurate species-level diagnosis since this technique was first approved by the U.S. Food and Drug Administration (FDA) in 2013 (11). However, a substantial proportion of hospitals reported relying exclusively on a single platform, most often MALDI-TOF MS (ACH: 36%, LTACH: 29%) or Vitek 2 (ACH: 10%, LTACH: 14%), which can only identify organisms already represented in their reference databases. Sole reliance on Vitek 2 is particularly concerning, given known challenges in accurately identifying rarer *Candida* species such as *Candida blankii and Candida famata* (7, 12). In addition, sole reliance on MALDI-TOF MS may be limited by the completeness of its spectral reference database; rare or emerging yeasts might be underrepresented, potentially leading to misidentification or failure to identify uncommon species (13). The low percentage of facilities routinely using chromogenic agar for primary specimens (ACH: 27%, LTACH: 32%) suggests an opportunity to improve laboratory practices as this low-cost and practical method can help differentiate *Candida* species and because bloodstream infections can have >1 species present (7, 12, 14).

Approximately two-thirds of ACHs and LTACHs had AFST available on-site or at an affiliated center, a higher proportion than reported in 2015 NHSN data on general ACHs (28%) (8). Compared with the prior study, automatic AFST without a clinician’s order was also more common, especially for bloodstream isolates (59% of ACHs and 55% of LTACHs vs <33% in 2015) (8). These increases are encouraging as reliance on off-site testing for AFST and requiring a clinician’s order (as opposed to using reflexive AFST) might slow turnaround, which could potentially delay appropriate therapy or limit opportunities for timely de-escalation from empiric echinocandin therapy to fluconazole (5, 8, 15). Although fluconazole remains the most commonly tested agent, we observed broader AFST coverage compared with the 2015 study, with more frequent inclusion of echinocandins and newer triazoles (8). This expansion might reflect the growing awareness of emerging resistance among *Candida* species.

Regarding *C. auris* screening, our findings complement those from a 2022 survey of Emerging Infections Network (EIN) members. However, direct comparison is challenging because the EIN survey represented a convenience sample of infectious diseases clinician responses from the same healthcare facilities could not be deduplicated, and participation was voluntary (16). In our study, PCR predominated over culture as the testing method (ACH: 52% vs 47%; LTACH: 73% vs 32%), contrasting with the prior study where culture was more than twice as common compared with PCR for healthcare facilities with in-house testing (67% vs 31%) (16). This finding could reflect differences in the survey design. It might also reflect increased PCR use due to its faster turnaround time compared with culture, which facilitates timely isolation and infection-prevention measures, as well as the availability of FDA-approved PCR assays for *C. auris* identification (16–18). The higher uptake of PCR and frequent use of surveillance testing upon admission in LTACHs (86%) might reflect the high risk of transmission in this setting and the need for rapid detection to prevent transmission among a medically complex patient population (2, 18). Because these data largely reflect practices before widespread availability of an FDA-cleared commercial PCR assay for *C. auris* colonization screening (cleared July 2024) (19, 20), future studies could evaluate whether such assays further influence implementation and standardization of screening practices.

Study limitations include that the NHSN PSC Annual Survey data were self-reported and could be subject to reporting or misclassification bias. In addition, we could not verify laboratory practices directly, assess test performance or turnaround times, or capture patient-level outcomes.

Nonetheless, we provided new benchmark data on national yeast identification practices and *C. auris* screening practices and updated information on AFST practices at ACHs and LTACHs. This information could support facilities in evaluating and strengthening their yeast identification, AFST, and *C. auris* screening practices to optimize patient care and enhance public health preparedness for emerging antifungal-resistant *Candida* species.

## Data Availability

Data from this report were obtained from the Centers for Disease Control and Prevention’s National Healthcare Safety Network, Patient Safety Component Annual Survey. Because these data include facility-identifiable information, they are not publicly available. Access to these data requires approval by the CDC according to NHSN data use policies (https://www.cdc.gov/nhsn/about-nhsn/dua.html).
